# Livestock impacts on an iconic Namib Desert plant are mediated by abiotic conditions

**DOI:** 10.1007/s00442-022-05177-w

**Published:** 2022-05-07

**Authors:** Jeffrey T. Kerby, Flora E. Krivak-Tetley, Saima D. Shikesho, Douglas T. Bolger

**Affiliations:** 1 Aarhus Institute of Advanced Studies, Aarhus University, Høegh-Guldbergs Gade 6B, 8000 Aarhus C, Denmark; 2 Department of Environmental Studies, Dartmouth College, Hanover, NH 03755 USA; 3 Department of Biological Sciences, Dartmouth College, Hanover, NH 03755 USA; 4 Gobabeb-Namib Research Institute, Namib Naukluft Park, Namibia; 5 Department of Biological Sciences, University of Cape Town, Cape Town, South Africa

**Keywords:** Exclosure, Drone, Desert, Water availability, Herbivory

## Abstract

Resolving the relative contributions of top-down versus bottom-up drivers of vegetation dynamics is a major challenge in drylands. In the coming decades, growing livestock populations and shifts in water availability will simultaneously impact many arid systems, but a lack of empirical data on plant responses to these pressures limits understanding of how plants will respond. Here, we combine ground and drone observations from an herbivore exclosure experiment to identify ungulate visitation patterns and their impacts on the cover and melon production of !nara (*Acanthosicyos horridus*), a large, long-lived desert plant in the hyper-arid Namib Desert. !Nara are of key ecological, social, and economic importance to Namib ecosystems and to the local Topnaar people. At our study site, we find that among native and domestic herbivores, free-ranging donkeys have the largest impact on !nara cover and melon production. !Nara cover was negatively affected by herbivores close to the desert-ephemeral river ecotone during a dry period, whereas !nara cover increased on all plants across the landscape during a wetter period, regardless of herbivore access. !Nara near the river channel and those protected from herbivores had more mature melons, particularly during the wetter period. At this site, the potential for conflict between Topnaar !nara melon harvesting and pastoral practices varies with a plant’s distance from the river and prevailing abiotic conditions. This work advances monitoring approaches and adds empirical support to the understanding that top-down and bottom-up regulation of plant dynamics varies with spatiotemporal context, even within landscapes.

**Supplementary Information:**

The online version contains supplementary material available at 10.1007/s00442-022-05177-w.

## Introduction

Livestock and other non-native ungulate populations are increasing globally (Hempson et al. 2015, 2017; Nyhus 2016; Svenning et al. 2016; Perino et al. 2019) with impacts on ecosystems that are complex and, at times, contentious. Much debate centers on whether non-native ungulates negatively impact ecosystem services or resilience, as with cattle grazing on semi-arid grasslands in the western USA (Fleischner 1994; Brown and McDonald 1995; Curtin 2002; Beschta et al. 2013; Svejcar et al. 2014) or the widespread ecological impacts of feral horse populations (Eldridge et al. 2020; Monsarrat et al. 2020). In contrast, trophic rewilding initiatives propose that, in some cases, non-native herbivores can functionally replace native herbivores, especially where native populations have declined or been extirpated (Lundgren et al. 2018).

Dryland regions are particularly sensitive to ungulate impacts (Beschta et al. 2013; Hempson et al. 2017). Characterized by low annual rainfall and high evapotranspiration, drylands cover over 40% of the Earth’s land surface and are currently home to 50% of the world’s livestock populations (Maestre et al. 2021). Both drylands and livestock populations are expected to increase in the coming decades due to the combined effects of climate change (Huang et al. 2016) and regional factors related to shifting economies and growing human population pressures (Oesterheld et al. 1992; Hempson et al. 2017; Wallach et al. 2018). The timing and magnitude of water availability strongly shapes trophic interactions in drylands (Noy-Meir 1973; Maestre et al. 2021), especially in deserts—arid and hyper-arid drylands which account for ~ 17% of global terrestrial environments (Maestre et al. 2021). High levels of endemism in deserts frequently make them regional diversity hot spots (Maestre et al. 2021) despite being host to fewer absolute numbers of plant and large herbivore species than other drylands.

Large plants make outsized contributions to desert ecosystems and economies, both as sources of critical food and/or habitat for other organisms (Fleming and Holland 1998; Franklin et al. 2016), and as culturally valued providers of goods and services for humans (Bidak et al. 2015; Goettsch et al. 2015), as with *Agave* and *Acacia* spp. (Springuel and Mekki 1994; Delgado-Lemus et al. 2014). Despite these important roles, the slow growth and long life spans characteristic of large desert plants can make their management and monitoring logistically challenging and expensive (Schweiger et al. 2020).

To evaluate the effects of herbivory on large, long-lived plants in deserts, it is imperative to understand plant responses in the context of water availability. Plant responses vary greatly across species and settings, spanning tolerance (Strauss and Agrawal 1999) and compensatory regrowth (McNaughton 1983), to the reallocation of resources to reproduction (Miller et al. 2006, 2008) or defense (Hanley et al. 2007). The evolution of plant defense strategies against herbivores is strongly shaped by resource environments (Stamp 2003), and theories like the Resource Allocation Hypothesis (Coley et al. 1985; Endara and Coley 2011) posit that in low resource environments, tolerance strategies of fast growth and recovery are comparatively costly for long-lived individuals, thus selection favors defensive herbivore resistance traits, like spines or thorns.

In deserts, rapid or unpredictable changes in resource conditions may mediate top–down pressures on plants both directly and indirectly. For example, McCluney et al. (2012) suggest that when surface water availability is low in an environment, herbivory will intensify as herbivores increasingly target surviving plants not only as energetic resources, but also as alternative sources for water (McCluney and Sabo 2009; McCluney et al. 2012). Many large desert plants persist in these low resource environments by storing or accessing water unavailable to herbivores, for example, via deep tap roots or by capturing water from fog. While these traits can ease water limitation, they may come at the cost of increasing a plant’s value to herbivores that may need to seek out plant derived water resources, especially during dry periods.

**Fig. 1:**
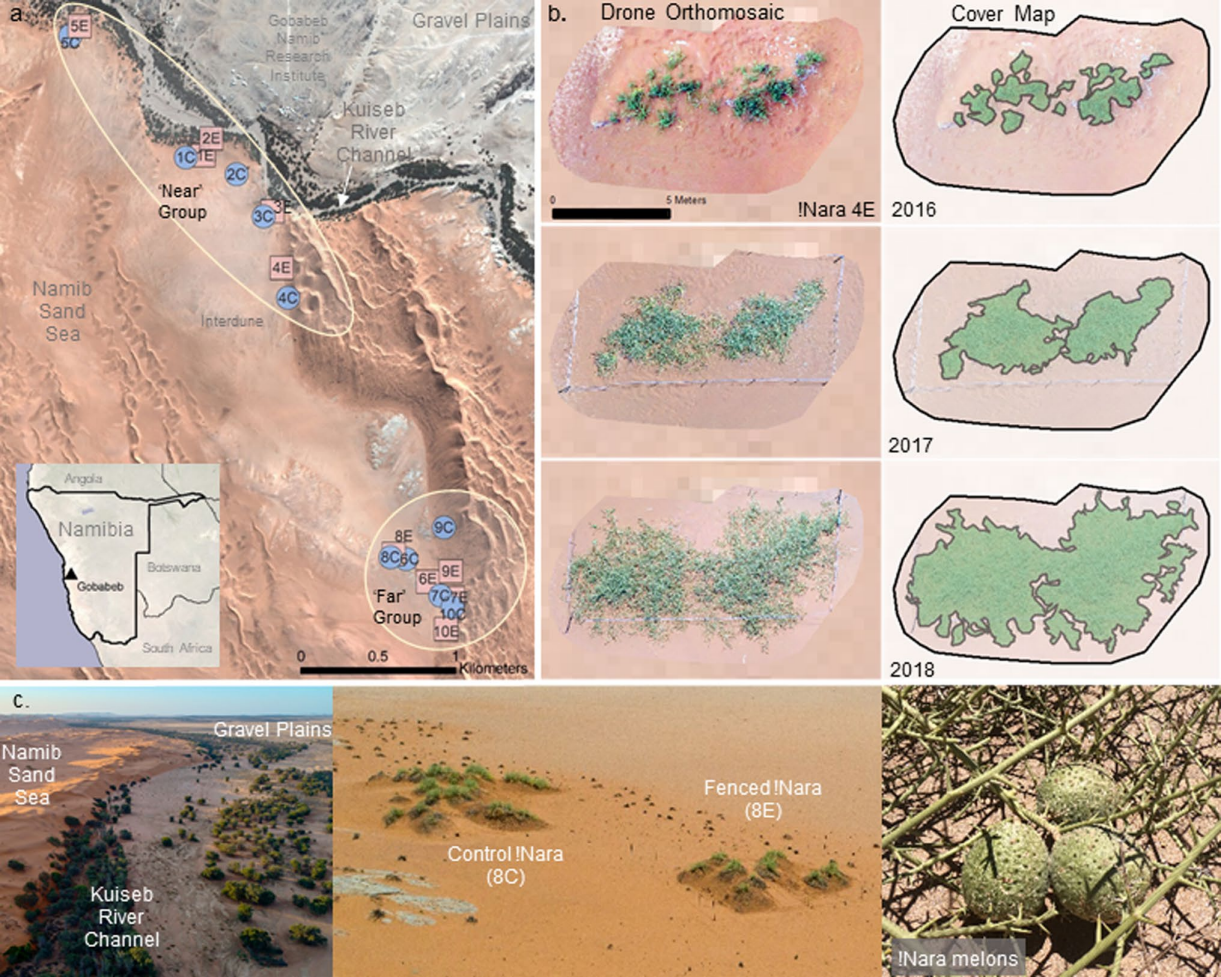
Study area and design. **a** Twenty female !nara plants were mapped with drones, initially in 2016, in the Namib Desert adjacent to the Gobabeb Namib Research Institute. The focal !nara plants were separated into two groups—those nearer to and generally at lower elevation by the Kuiseb River and the riparian aquifer (‘near’), and those inland over a kilometer away from the Kuiseb River beyond the high dunes (‘far’). Preliminary observations and local knowledge suggested these regions represented opposing ends of a livestock pressure gradient, with higher browsing from large herbivores near the riverbed. **b**, **c** Ten of the twenty plants were fenced off to exclude large herbivores. Monthly ground observations of melon production and herbivory signs were paired with annual drone-generated orthomosaics that were digitized to measure the extent of vegetation cover. Orthomosaics and cover maps of all !nara plants for all years are located in Fig. S1

Here, we focus on the effects of ungulate herbivory on !nara (*Acanthosicyos horridus*), a large, long-lived plant species of key ecological, cultural, and economic importance at our study site and throughout its endemic range in the Namib Desert (Henschel et al. 2004). !Nara is a leafless and dioecious cucurbit that forms sprawling, sandy hummocks that can rise to over 5 m in height and cover over a thousand square meters (Kartusch and Kartusch 2008; Gerber 2018). They mainly grow in and around the ephemeral rivers that cross the hyper-arid Namib Desert, with a distribution that spans western Namibia, southwestern Angola and northwestern South Africa (Klopatek and Stock 1994). Along Namibia’s ephemeral Kuiseb River (Fig. 1), !nara also occur in the adjacent interdune valleys of the Namib Sand Sea, part of the Namib-Naukluft National Park and an IUCN World Heritage Site (Seely 2012).

!Nara are of particular conservation interest due to their limited distribution and their economic and cultural importance to the Kuiseb Topnaar (or ≠ Aonin) people. The Topnaar are a Khoekhoegowab-speaking community whose historically documented presence along the lower Kuiseb River dates back centuries (Kinahan 2017). Approximately 350 Topnaar live in small, rural settlements along the Kuiseb and partially rely on livestock husbandry and !nara melon harvesting (Dieckmann et al. 2013), a long-standing practice that also holds a prominent place in Topnaar culture (Dentlinger 1977; Gruntkowski and Henschel 2004; Moser and Henschel 2004; Mizuno and Yamagata 2005). Pulp and seeds of harvested melons are processed for local consumption, or are sold to local enterprises that press oil from the seeds which they use to make a variety of food and cosmetic products, increasingly for the tourist market (Cheikhyoussef et al. 2017; Chinsembu and Chinsembu 2020).

Topnaar livestock holdings include goats, sheep, cattle and donkeys. Cattle and donkeys are allowed to free range alongside native herbivores, such as gemsbok (*Oryx gazelle*) and springbok (*Antidorcas marsupialis*) throughout the year. Small stock are penned at night and often attended by a herder to deter predators (pers. obs.; pers. comm.). For much of the year, preferred ungulate forage resources are primarily found in or adjacent to the riparian woodlands of the Kuiseb (Shiningayamwe 2019; Morgan et al. 2021; Fig. 1), particularly the pods of *Vachellia erioloba* and *Faidherbia albida* which are consumed after they fall to the ground. Various grasses and other small forage species also become available and/or particularly palatable in the interdune areas for a few weeks following infrequent rain events (Seely and Louw 1980). !Nara are found both in and around the riparian woodlands and, in some cases, several kilometres into the interdune landscape.

!Nara deter herbivory and defend against excessive transpiration via dense, photosynthetic stems and spines formed from modified leaves (Fig. 1c). Water relations of !nara are not fully documented, but existing research indicates that they may acquire water from multiple sources: groundwater, rainfall events, and potentially from fog. !Nara access groundwater via their long, highly vascularized tap root (Klopatek and Stock 1994; Kartusch and Kartusch 2008; Gerber 2018), but it has been suggested that the magnitude of flowering and melon production may partially depend on rare, stochastic rainfall events (Klopatek and Stock 1994; Gerber 2018). Experimental and morphological studies suggest that !nara may absorb or channel fog water via their stems and adventitious roots (Kartusch and Kartusch 2008; Gerber 2018), though isotopic analyses have found no support for this (Soderberg 2010). Unlike rainfall, fog is one of the few consistent water sources in the Namib and it is widely relied upon as a water source for Namib insects and plants (Soderberg 2010), although its frequency declines with distance inland from the coast (Mitchell et al. 2020; Weathers et al. 2020). Given their potential use of multiple water sources, !nara should be considered either a semi-obligate, or a semi-facultative, phreatophyte, using the terminology of Hultine et al. (2019).

Livestock herbivory on !nara is commonly observed, and thus Topnaar use of livestock and !nara melons are potentially in conflict (Henschel et al. 2004). The impacts of livestock and wild ungulates on !nara growth and melon production are assumed primarily from observational and anecdotal reports, but the characteristics of !nara and its environment suggest that these effects may range from strong to minimal. As most plant species exhibit a reduction in growth and/or reproduction when subject to herbivory (Hawkes and Sullivan 2001), we expect that plants experiencing routine exposure to livestock may have reduced melon output and/or stem production. Domestic livestock in the lower Kuiseb River region are water supplemented, number in the hundreds (roughly 300–400 cattle, 100 donkeys, 300 + goats) (Shiningayamwe 2019) (unpublished data), and occur at higher densities and more consistently through seasons and droughts than wild herbivores (pers. obs.). Thus, !nara could be exposed to higher levels of herbivory now than in the past, calling into question their ability to deter, resist or tolerate domestic herbivory. In contrast, as phreatophytes, !nara may not be as water limited as the low rainfall in this region of the Namib would suggest. Phreatophytes in desert environments are capable of responsive and sustained vegetative growth (Hultine et al. 2019); thus, relative to other plants in the Namib, !nara may be able to compensate for herbivory.

Here, we use a novel combination of ground- and remote-sensing data from a multi-year large-herbivore exclusion experiment to evaluate how herbivory affects !nara growth and melon production in parts of a landscape with differing herbivory intensity and ground water availability. Specifically, we set out to: 1. identify patterns of native and domestic large-herbivore visitation to !nara across the landscape, and 2. to quantify large-herbivore impacts on !nara growth and melon production across differing local top-down and bottom-up contexts.

## Methods

### Study site and experimental design

This study was conducted between 2016 and 2018 around the Gobabeb Namib Research Institute (23.566° S, 15.040° E) in the Namib-Naukluft National Park, central Namib Desert, Namibia (Fig. 1). The central Namib Desert at Gobabeb is characterized as hyper-arid desert with low rainfall (25 mm yr^−1^) and extreme temperatures (2–43 °C)(Lancaster et al. 1984) and comprises three ecosystems: the Namib Sand Sea (Aeolian dunes), the ephemeral Kuiseb River channel (linear oasis), and gravel plains (Fig. 1a, c). !Nara populations are found in or along the Kuiseb River and at the base of dunes south of the Kuiseb, but they do not occur on the gravel plains north of the Kuiseb.

Twenty female !nara plants were selected for observation in November 2016 in the interdune valley closest to Gobabeb (Fig. 1a). We used only females, since one of the main motivations of the study was to understand the effects of herbivory on melon production. Furthermore, female plants in dioecious species may be more vulnerable to increasing aridity and other consequences of climate change due to their higher resource requirements (Hultine et al. 2016). Plants were selected in two regions, near (10 plants) and far (10 plants) from the Kuiseb River channel, and they were assigned arbitrarily to experimental or control groups (Fig. 1a, Fig. S1). All plants were separated by tens to hundreds of meters from any neighboring conspecifics (Fig. 1). The ‘near’ plants were on average 230 (± 155) meters from the river channel (straight line) with an average elevation of 408 (± 12.5) m, while the ‘far’ plants were on average 1430 (± 125) m from the river channel with an average elevation of 428 (± 5) m. This partitioning was done for several reasons. First, it matched the existing distribution of female !nara at the site. Second, a priori*,* we expected that the combined distance above *and* away from the river would affect ground water availability (Eppley and Wenk 2001; Henschel et al. 2004; Morin et al. 2009; Grodek et al. 2020) and the intensity of herbivory by wild and domestic ungulates that prefer foraging in and around the shaded and more densely vegetated river channel (Kok and Nel 1996; Moser-Nørgaard and Denich 2011). The !nara further from the river are on average 20 m higher above the riparian-fed water table centered around the river channel, and thus should have reduced water availability via their taproots, particularly during dry periods when the local water table in this part of the landscape drops (Klopatek and Stock 1994; Grodek et al. 2020). The distribution of these ‘far’ !nara in the interdune of the adjacent Namib Sand Sea corresponds with the distribution of paleo-channels carved into the bedrock by earlier flow paths of the Kuiseb River that are now buried by sand (Muller 2004; Paillou et al. 2020). Hydrological analyses and field sampling indicate that these channels likely trap groundwater for a time period after recharge, and in some cases connect and/or drain into the Kuiseb aquifer (Klaus et al. 2008; Paillou et al. 2020). Like other studies, we assumed that ‘far’ !nara probably occur over paleo-channels, like those partially mapped in Muller (2004) and Paillou et al. (2020).

Following !nara selection, seven-strand steel fencing approximately 1.5 m in height was placed around all of the foliage of each experimental plant and its underlying sandy hummock. This fencing design is common on livestock farms in Namibia and is sufficient to exclude most large ungulates as long as it is adequately maintained. It does not exclude small herbivores, which are able to slip unimpeded through the fencing and thus equally access all !nara in the study.

### Ground-based measurements

To measure the intensity of large herbivore use of !nara hummocks we used a fecal accumulation rate approach (Campbell et al. 2004; Skarin et al. 2020). All dung found around each plant was identified, counted, and removed from the hummock monthly. For experimental plants, this meant all dung found within the fenced area, while for controls it included all dung on the hummock and within a meter of its base. Dung was identified to species: donkey, cow, gemsbok, and small ungulate (could not differentiate sheep/goat/springbok). The abundance of medium and large mature melons (> 12 cm diameter) was counted each month, with annual peaks evident in November–December during the course of our study (Fig. S2). We used November counts in this study, as this corresponds with the end of the ripening season/pre-harvest. Based on conversations with the local community, we understand that none of the focal !nara were subjected to harvesting during the study period.

### Abiotic and satellite measurements

We characterized the long-term, landscape-scale abiotic and biotic context of the site and study period using monthly rainfall records gathered by sensors at the Gobabeb-Namib Research Institute and monthly measures of the normalized difference vegetation index (NDVI) of the interdune area where this study took place. NDVI is a spectrally based measure of landscape ‘greenness’ derived from reflectance in the near infrared and red regions of the radiometric spectrum that correlates with vegetation properties like the leaf area index (LAI) and the fraction of absorbed photosynthetically active radiation (fAPAR) (Myneni and Williams 1994; Pettorelli et al. 2005). In low vegetation environments with high surface reflectance, like the Namib Desert, the dynamics of NDVI experience lower signal to noise ratios (Myneni and Williams 1994), but time series can still accurately reveal productive periods and the relative magnitude of landscape-level vegetation activity (Mitchell et al. 2020; Maestre et al. 2021; Morgan et al. 2021). NDVI data were measured using NASA’s MODIS MOD13Q1 v006 collection (Didan 2015) of 16-day, atmospherically corrected 250 m vegetation index product resampled at monthly time intervals over the study site using Google Earth Engine’s online application programming interface (Gorelick et al. 2017).

### Drone image capture and processing

Large plants (hundreds to thousands of m^2^) are logistically challenging to measure systematically, accurately and efficiently. To overcome these challenges, and to avoid disturbing the focal research plants, we used drone-mounted cameras to capture a series of overlapping nadir and oblique digital photographs of every study !nara each November from 2016 to 2018. Photos in 2016 were captured with a 12-megapixel Canon S100 series CMOS digital camera mounted on a 3DR Iris + quadcopter (either in RGB or using a camera with the near infrared filter removed), and photos from 2017 and 2018 were captured in RGB with the 20 megapixel CMOS digital camera integrated into the DJI Phantom 4 Advanced quadcopter.

Automated and manual flight plans were flown at altitudes varying from 10 to 30 m (relative to size and landscape position of plant) to produce imagery sets with > 80% fore and side overlap across the entire plant. A digital orthomosaic was produced for each plant each year with a spatial grain of < 2 cm using standard workflows in Agisoft Photoscan Pro, v1.4.3. Orthomosaics from 2017 were scale validated using within-scene markers (2 m pieces of wood planking and ground measurements between geostationary landscape features) and then used as the reference coordinate system for the study. Orthomosaics from 2016 and 2018 were generated using similar procedures and were then geo-referenced with 2017 models in ArcMap 10.3 using stationary landscape reference points in common with the 2017 orthomosaics. After spatial alignment, each orthomosaic was clipped to an extent corresponding to the perimeter of each focal !nara hummock and re-projected into UTM coordinates.

### Plant cover and melon analyses

!Nara vegetation cover was measured each year for each plant by manually digitizing the two-dimensional cover perimeter of green (i.e., living) !nara patches in each orthomosaic (Fig. 1) in Arcmap 10.3 with sub-decimetre tolerances, and then by calculating the internal area of these digitized polygons using the sf package (Pebesma 2018) in R (R Core Team 2019). Methods of supervised and unsupervised pixel-based classification for cover mapping were also explored, but these approaches resulted in inconsistencies across inter- and intra-annual time series due to differences in illumination conditions, ground litter, substrate composition, and camera models (Fig S1). We consider our methods a conservative correlate of biomass, but a much more accurate and less destructive way of measuring dynamics of whole !nara growth patterns than previous ground-based approaches.

To measure the change in plant vegetative growth from year to year while accounting for differences in plant size, we calculated the between-year difference in area of green cover for each year of the study (2016–2017 and 2017–2018) and standardized this by the total area of green cover measured on each plant in the previous year (2016 or 2017). We standardized melon production by dividing the number of mature fruits counted in November on each plant by the total area of green cover measured on each plant that year. To explore for potential plant size-related confounding effects, we looked for relationships between each response variable and the surface area of each !nara hummock (hummocks grow outward and upward over decades as sand infills stems which are then buried, thus this metric captures elements of both above and belowground biomass), but found no consistent relationships across plants (Fig. S3).

### Statistical analyses

All statistical analyses were conducted using R (R Core Team 2019). We used two-way ANOVA to test for effects of large ungulate herbivory (fenced vs unfenced) and water availability (near river/aquifer vs far from river/aquifer) on proportional change in plant green cover (as described above) from 2016–2017 and 2017–2018, and melon productivity in November (Austral summer) in 2017 and 2018. In the case of significant interactions, we followed up with post hoc Tukey’s honestly significant difference (HSD) tests to identify differences between groups.

## Results

### Rainfall and landscape greenness

**Fig. 2:**
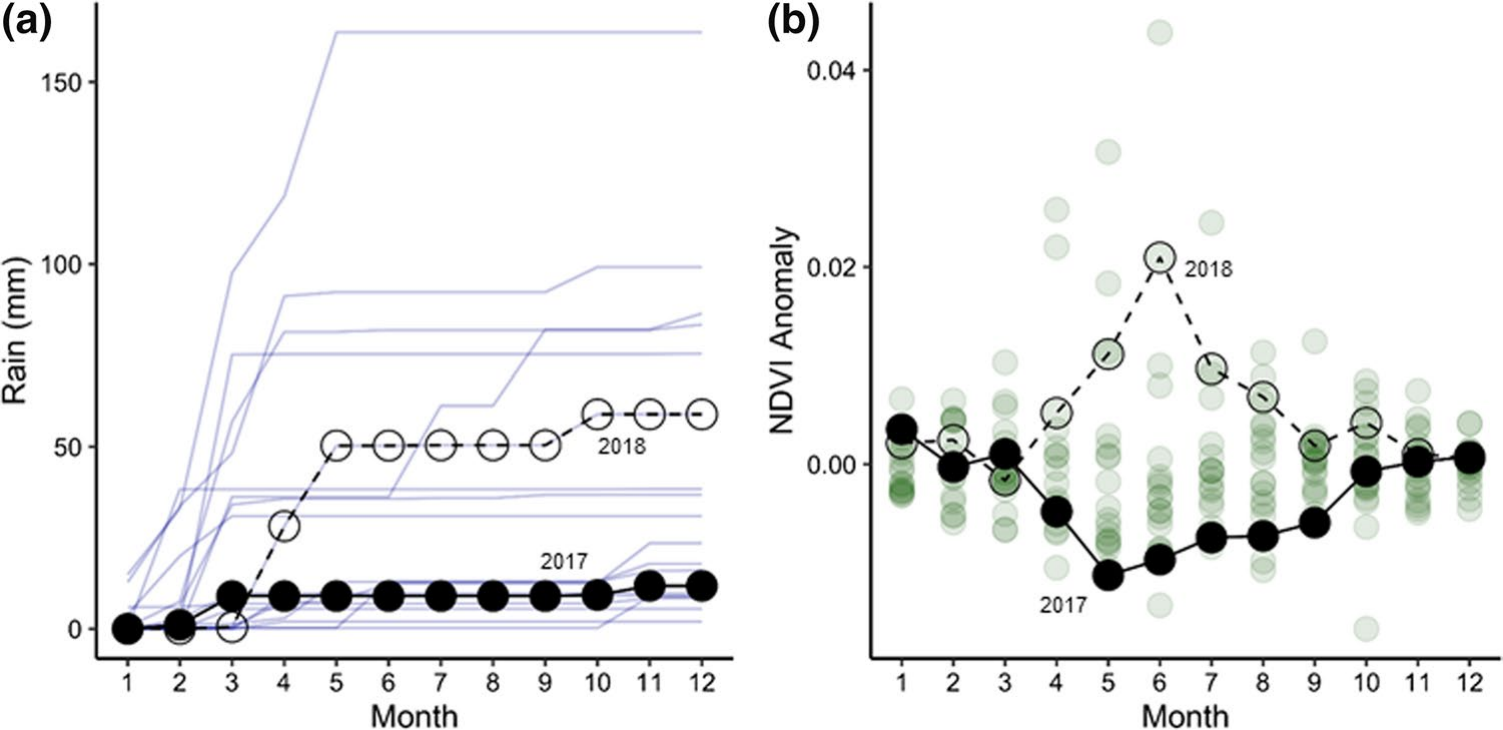
Patterns of annual rainfall and NDVI of the study area. **a** Cumulative rainfall (mm) measured at the Gobabeb Namib Research Institute meteorological station for each year from 2000–2018 (pale blue lines), and **b** NDVI (unitless) anomalies each month from the 2000–2018 NDVI monthly means (pale green circles). The drier, less green year of the experiment is symbolized with filled circles (2017) and the rainier, greener year is symbolized with open circles (2018)

Rainfall is sparse in this region of the Namib, with an annual median of 20.7 mm and mean of 41.3 (± 9.8 S.E.) mm over the period of 2000–2018 (Fig. 2a). Rainfall in 2017 was approximately 50% the 19-year median (the period of corresponding MODIS NDVI availability), whereas 2018 rainfall was nearly 300% greater than the median of this period (Fig. 2a). Only a single rainfall event (~ 5 mm) occurred in 2017 (in March), whereas large (> 20 mm) and moderate (between 8 and 20 mm) rainfall events occurred in April, May, and October in 2018 (Fig. 2a). Positive landscape greenness (NDVI) anomalies corresponded with or slightly lagged rainfall events (Fig. 2b). The annual mean NDVI of the interdune is 0.10. In 2017 there were only four months above the long-term average monthly landscape greenness, whereas 11 of 12 months in 2018 exceeded the 19-year monthly greenness average, including one month in the 97th percentile of landscape greenness among all months in the 19-year satellite record (Fig. 2b).

### Large herbivore visitation

**Fig. 3:**
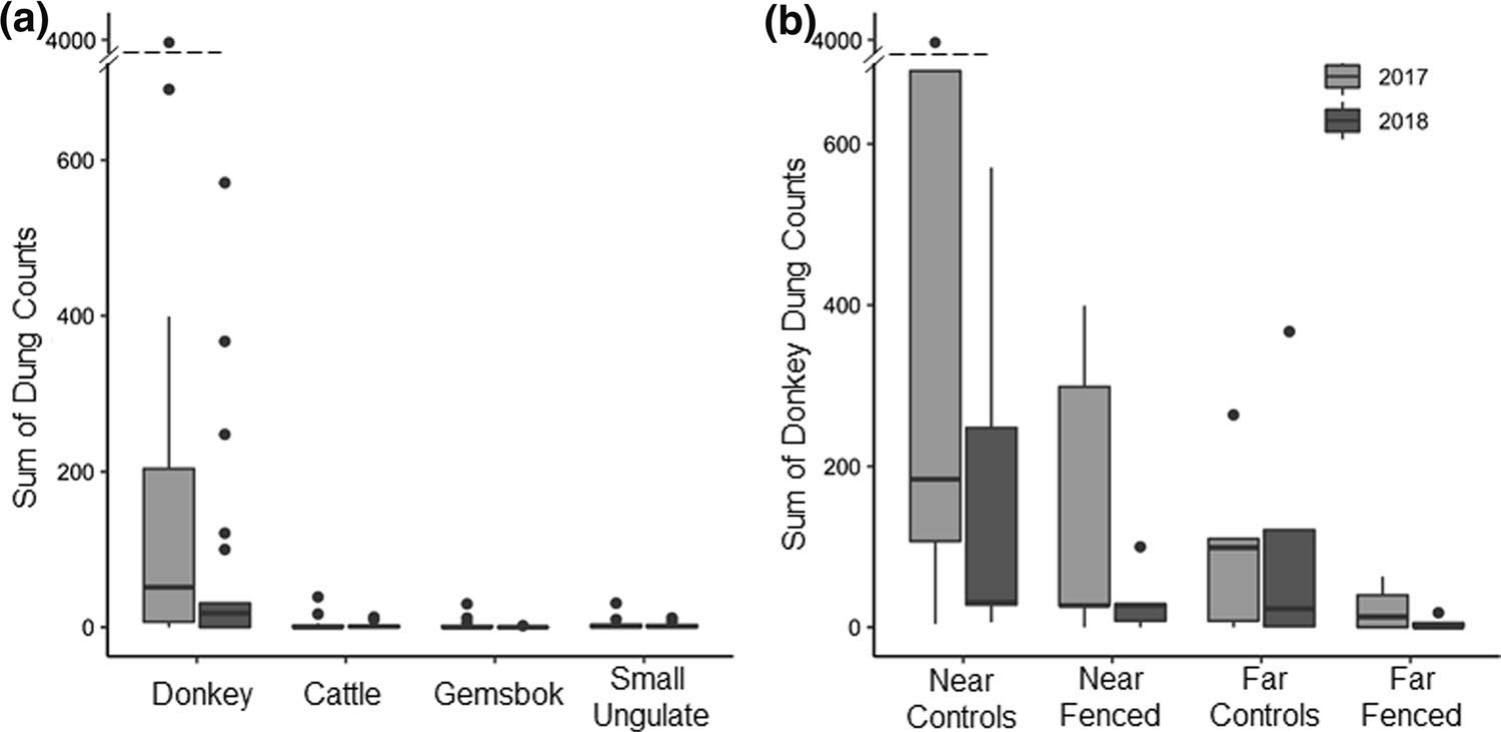
Annual cumulative herbivore fecal counts across study plants. **a** Donkey dung was by far the most common type of dung found at any site in either year. Due to difficulties in distinguishing springbok, sheep and goat dung, they are combined as the Small Ungulate category. **b** Fenced plots consistently kept donkeys and other large herbivores out, except on a few occasions when fences were breached but then mended within a month. In both years there was a general pattern of reduced herbivore dung on the plots located further from the Kuiseb River

Large herbivores frequently visited !nara plants, and donkeys were the predominant visitors (96% of the 8497 dung deposits); others included cattle and native herbivores like gemsbok and springbok (Fig. 3a). The exclosure treatment successfully reduced large herbivore visits (Fig. 3b), although some dung was noted on enclosed hummocks due to rare breaches of the fences and also presumably due to dung being blown into the exclosures or exposed by wind in the case of old dung previously covered by sand. !Nara hummocks near the river showed evidence of much more intensive visitation; donkey dung deposition was 5–10 × higher on hummocks near, compared to far from, the ephemeral river (Fig. 3b).

### Growth and melon production

**Fig. 4:**
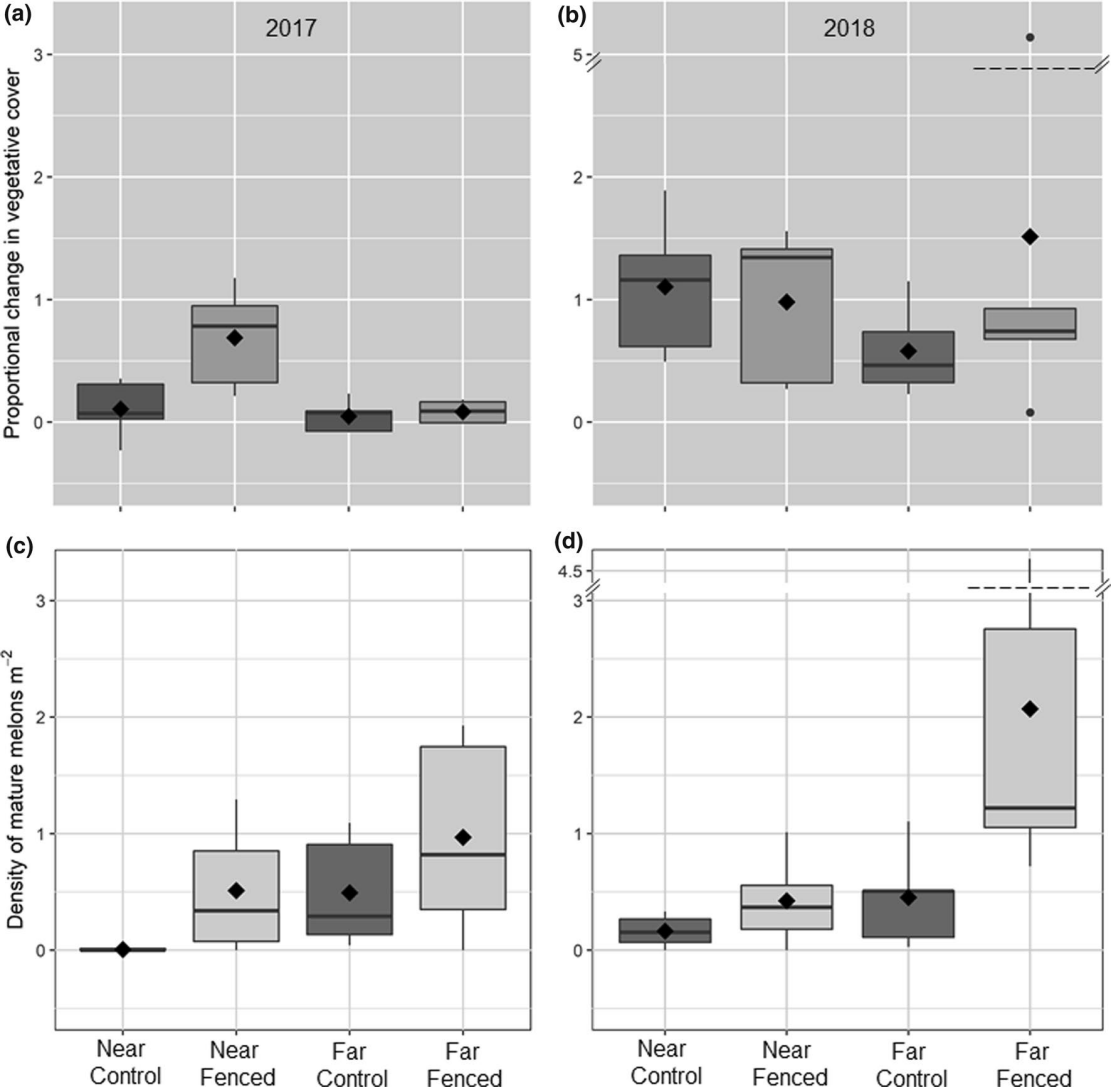
Boxplots of median and mean (diamond symbols) !nara growth and melon production over the two study windows. Over the arid 2017 period, !nara on herbivore-visited plots near the river (i.e., the near controls) showed (**a**) little extent-cover change or (**c**) melon output, whereas both control and exclosure (i.e., fenced) plots far from the river showed higher melon output but no differences in cover change in that time period. All !nara extent-covers grew proportionally larger in the wetter 2018 period (**b**), but there were clear herbivore effects on melon output (**d**). Across all years, melon density was greater on herbivore excluded plots relative to controls within each location block

The growth of !nara plants in the drier 2016–2017 period varied among treatments (control and fenced) and proximity to the ephemeral river/aquifer (near and far)(Table 1, Fig. 4a). During this period, plants protected from herbivory (fenced) near the river grew proportionally more than browsed plants near the river (*t* = − 3.68, adjusted df = 16, *P* = 0.01) but this was not observed far from the river (*t* = − 0.23, adjusted df = 16, *P* = 0.99), where growth patterns in both fenced and browsed plants were comparable to the browsed plants close to the river (Fig. 4a; all *P* > 0.98). In the wetter 2017–2018 period, all !nara plants grew larger, with no consistent differences among treatments and locations (Table 1, Fig. 4b). There were no consistent trends in proportional growth of !nara relative to hummock size over the duration of the study, although there was more variability in smaller individuals, which were more common in the dataset due to the size distribution of plants growing in the study valley (Fig. S3).

**Table 1:** ANOVA results for proportional change in live !nara vegetation cover during 2016–2017 and 2017–2018 and peak density (as measured in late November) of mature melons from 2016–2017 and 2017–2018

Green Cover		*F*_(1,16)_	*P*
2016–2017	Treatment	7.66	**0.01**
	Location	8.77	**0.01**
	Treatment × Location	5.96	**0.03**
2017–2018	Treatment	0.65	0.43
	Location	0.00	0.99
	Treatment × Location	1.10	0.31

Melons			
2017	Treatment	3.86	0.07
	Location	3.57	0.08
	Treatment × Location	0.00	0.95
2018	Treatment	5.94	**0.03**
	Location	6.30	**0.02**
	Treatment × Location	3.11	0.10

Total sample size for all tests was *n* = 20 experimental plants

We also observed significant variation in melon production over the course of the study. Plants far from the Kuiseb River consistently had more mature melons per unit area than those close to the river, and in both locations, plants protected from herbivores produced proportionally more melons than controls that were left unfenced (Fig. 4c, d). These patterns were only significant in the wetter second year of the experiment (*P* = 0.02–0.03; Table 1) with similar, albeit marginally non-significant patterns in the drier first year (*P* = 0.07–0.08). Over the course of the study, there were no consistent patterns in !nara melon production per unit green cover in relation to overall hummock size, although maximum melon production per unit vegetation tended to occur in smaller !nara (Fig. S3).

## Discussion

Our results indicate that trophic impacts from large herbivores on !nara are sufficient to limit cover growth and reproductive output under certain conditions of water scarcity and browsing pressure, and that domestic donkeys appear to be driving this herbivore effect. Local rainfall and groundwater both influence when and where !nara dynamics are sensitive to herbivory. !Nara near the river have greater access to water but suffer high donkey herbivory, whereas !nara far from the river suffer lower levels of herbivory but are more likely to be water limited.

Near the Kuiseb River, donkey herbivory is sufficiently intense to limit growth and, to some extent, melon abundance in a dry year (2017a, c, Fig. 4, Table 1). In a high rainfall year, !nara by the river seem to be able to deter or compensate for herbivory, with no effect on growth or melon output evident. Further from the river, donkey visitation was much lower (Fig. 3b). During a dry year, there was little vegetative growth and no detectible herbivore effect on growth here, but herbivory did affect melon abundance. In the wet year, these !nara far from the river increased in cover size and had higher melon density (Fig. 4b, d). Again, herbivory had no effect on growth, but did affect reproductive output at this site. These results suggest the level of herbivory far from the river was not sufficient to limit !nara expansion, but was sufficient to limit the production of mature melons.

Although we observed differences in vegetative growth between treatments in 2018, the treatment and treatment x location factors were not significant. This lack of significance is likely attributable the high variation in growth among the far controls in 2018 (Fig. 4b). Some plants showed vigorous growth while others exhibited no response (Fig. S1). This could be due to localized differences among plants in site quality or belowground water availability. Herbivory may have had some effect on growth of far plants in 2018, which is consistent with the higher visitation by donkeys in 2018 compared to 2017 (Fig. 3b).

Herbivory had a more consistent effect on melon density than it did on vegetative cover change, though these effects may be both direct and/or indirect. While frugivory by donkeys directly impacts melon density, their herbivory on photosynthetic tissues may also indirectly reduce resources available for fruit production, potentially leading to a shift in resource allocation from reproduction to growth (Wenk and Falster 2015). Unfortunately, we are unable to differentiate the relative importance of these direct and indirect effects. The stronger impact of herbivory on melons than cover could be indicative of donkey’s preferentially foraging on the melons relative to the stems. !Nara melons are distinct from most other forage items in the desert, in that they are concentrated and abundant sources of water and carbohydrates (Klopatek and Stock 1994). Donkeys and many other species, especially jackals (Shikesho 2020), appear to have a preference for !nara melons independent of environmental conditions. Henschel et al. (2004) observed that in dry conditions, donkeys will sometimes consume, or simply remove and/or damage, many dozen !nara melons and flowers in a single foraging bout, sometimes stripping an entire plant of its reproductive tissue. At this site, herbivory represents a greater threat to !nara located near the river relative to those located further into the interdune despite the advantages of consistent water availability. !Nara near the river were capable of vegetative growth even in a low rainfall year (Fig. 4a). These plants are also subject to intense donkey herbivory that is capable of limiting vegetative growth (Fig. 4a). However, in the wetter year, !nara compensated, at least in aboveground cover expansion, for this high level of herbivory. We note that two plants in our study located near the river had been eaten down to nubs prior to the installation of fencing around one of them (see plant E5 in Fig. S1). Our observations of these control and fenced individuals suggest that plants that have experienced extreme browsing pressure are less able to immediately respond to herbivore removal than other, only moderately browsed plants (see plant E5 in Fig. S1), though we are unable to adequately evaluate this hypothesis with the current study.

Eppley and Wenk (2001) proposed that groundwater may be more limiting for !nara situated higher above the riparian aquifer. If, as might be expected during dry periods, evaporation and drainage cause the water table in paleo-channels in the interdune to drop earlier than the lower lying, flood-recharged Kuiseb River channel (Klaus et al. 2008), a landscape-scale gradient in water limitation will emerge. The complex hydrology of this system warrants further investigation (Paillou et al. 2020), but our results are consistent with the possibility that both herbivory and a gradient in water availability are capable of directly limiting !nara growth within this landscape—especially during dry periods.

Shifts in herbivore foraging behavior due to changes in environmental conditions may further explain why !nara are less successful at deterring herbivore browsing on their stems during dry periods. Klopatek and Stock (1994) anecdotally report that native herbivores increasingly target !nara during times of low rainfall when other local forage species, such as dune grass (*Stipagrostis sabulicola*), desiccate, become less palatable, or disappear (e.g., Fig. 2). In arid environments, herbivory effects are expected to intensify as water availability decreases, with herbivores increasingly targeting surviving plants as both sources of water and energy until conditions improve, the herbivores die, or they move elsewhere (McCluney et al. 2012). Clearly, !nara’s anti-herbivore defenses are insufficient to inhibit all donkey herbivory during dry periods, even for plants near the river (Fig. 4a, c). Unlike native gemsbok at this site, which will move elsewhere or die during intense dry periods (Hamilton III et al. 1977), donkeys are provisioned with well water by humans. This creates opportunities for populations to be maintained at densities not fully regulated by environmental conditions, and thus exert a trophic pressure outside of the context under which !nara’s anti-herbivore defenses evolved, even in relation to other equids that may have been more common in this system in the past, such as zebra. The ecological and conservation implications of herbivore water provisioning are complex and multifaceted (Krausman et al. 2006), and evaluating species-level herbivore impacts should account for differential access to water among domestic and wild herbivores. Large predators may also indirectly impact herbivore foraging behavior (Smith et al. 2019), though the low predator densities in this system and the time-scale of this experiment make them an unlikely driver of the herbivore impacts reported herein.

If donkeys have meaningful impacts on !nara melon production, there may be instances where Topnaar pastoral practices threaten their own !nara harvesting outcomes. Better understanding these tradeoffs can empower resource users to make informed choices. We have identified that donkey impacts on !nara are variable and are influenced by environmental conditions at this site. However, we caution against extrapolating from our results to draw definitive conclusions about Topnaar resource-use tradeoffs and decision making from this single study. The !nara the Topnaar harvest occur in a variety of settings in addition to the interdune environment and study site addressed here. In particular, many !nara fields occur in the Kuiseb riverbed itself. These !nara may have sufficient access to water to compensate for herbivory. Furthermore, !nara in that topographically protected setting do not grow on hummocks and have a more compact morphology that seems to limit herbivory to terminal branches. Describing these dynamics will require additional research across a broader and more representative range of environmental, social, and ecological contexts found throughout the full length of Kuiseb River. Our findings do, however, provide novel experimental support to earlier observational reports about the importance of donkey impacts on !nara at this site and elsewhere in the Kuiseb River Delta (Henschel et al. 2004). Previous dissections of hundreds of donkey droppings from around Gobabeb and the Kuiseb Delta region revealed !nara seed fragments were present in 43% of samples, but all were damaged and none contained a viable embryo (Muller 2004). Thus, in contrast to jackals (Shikesho 2020), it is unlikely that donkey herbivory provides fitness boosting seed dispersal benefits to !nara.

Any potential negative effects of donkeys on !nara must also be considered in the context of the positive services donkeys provide to local human and wildlife communities. In many settings, donkeys are used for transport, food, farming, and potentially as critical fallback sources of income, especially for women (see Maggs et al. (2021) for examples from Ghana). Donkeys may also perform important functions in the broader ecosystem in parallel with or in place of native herbivores. For example, similar to gemsbok (Hamilton III et al. 1977), donkeys are capable of engineering desert water availability by well digging that positively affects other species (Lundgren et al. 2021). Although donkeys destroy !nara seeds when they are consumed, their habit of breaking open and leaving melons likely benefits many smaller mammals and insects (Shikesho 2020). While in some regions of Africa, demands for donkey skins from China have driven commodity markets and rapid increases in donkey populations (Wallach et al. 2018), to our knowledge this has not occurred in Namibia.

!Nara may live for centuries (Klopatek and Stock 1994), and while recruitment and mortality events are rare in these populations, our results have implications for understanding risk factors in their population dynamics. At this site, the presence of !nara far from the river appears dependent on two factors: the availability of groundwater due to paleo-channels and a low level of herbivory—at least some of the time. Our data indicate the conditions there only periodically support !nara growth. Thus, it is questionable whether these plants could persist in the face of sustained increases in herbivory levels, or if they experienced prolonged reduced water availability.

Large, long-lived plant species are underrepresented in landscape-scale ecology and conservation planning efforts relative to their importance to ecosystems and societies (Schweiger et al. 2020), especially in deserts. Drone monitoring offers solutions to some of the logistical challenges that contribute to this pattern and represents a scalable approach to the study !nara or other large, long-lived plants elsewhere. Ground surveys of large, well-defended plants are time consuming, imprecise, and potentially destructive (Bråkenhielm and Qinghong 1995), and are very difficult to replicate or compare through time. Drone imagery allows for systematic and non-destructive monitoring of large individuals and landscape-scale experiments through time, and results in a form of archival data (i.e. fine gain imagery) suitable for future analyses or applications beyond the scope or objectives of the initial research design, particularly as processing methodologies become better standardized and evaluated across systems (Cunliffe et al. 2021). For plants like !nara, whose population and recruitment cycles extend well beyond those of a single researcher’s career (Klopatek and Stock 1994), having these quantitative, long-term data are themselves of great conservation value. More generally, herbivore exclosure experiments, especially those including a measure of herbivore visitation pressure, are critically underrepresented in deserts, despite being one of the strongest and most widely used methods of evaluating large herbivore impacts on plant ecology (Jia et al. 2018; Forbes et al. 2019).

The threats of increased herbivory and shifting water availability faced by !nara mirror general threats faced by plant species in drylands globally. In the coming decades, as livestock populations grow and water competition increases in global drylands (Güneralp et al. 2017; Hempson et al. 2017; Shukla et al. 2019), the population dynamics and subsequent conservation status of many plants in arid regions will be affected. Despite being a charismatic species of cultural and economic importance, !nara remain among the 94% of global plant species that have not been evaluated against current IUCN Red List Criteria (2021). While clear threats to some dryland plant taxa, such as cacti, are well assessed and established, this remains the exception rather than the rule in arid regions (Goettsch et al. 2015).

Understanding the ecological conditions that affect plant population dynamics is critical for managing these populations under climate change. For !nara, projections through the end of the twenty-first century predict a Namib that is hotter (Engelbrecht and Engelbrecht 2016), with increased evaporation, and lower rates of aquifer recharge (Niang et al. 2014) and growing water extraction pressures from urban and industrial sources. In recent decades, rainfall has become more variable at this study site (Mitchell et al. 2020), and has increased more generally around ephemeral rivers across Namibia (Rohde et al. 2019). The regional climate around the Namib Desert is driven primarily by the cold water of the Benguela upwelling, which restricts rainfall (Eckardt et al. 2013) and shapes a fog gradient that penetrates roughly 100 km inland (Rohde et al. 2019). !Nara’s broader distribution roughly coincides with this fog belt (Klopatek and Stock 1994; Gerber 2018), yet fog frequency and inland penetration are expected to decrease as the Namib warms (Haensler et al. 2011). Thus, all three potential water sources for  !nara : rainfall, aquifers, and fog, are expected to change in the years ahead. This local study suggests the effects of these changes on regional !nara populations will be variable across sites and landscapes, and be mediated—rather than dominated—by trophic context.

## Supplementary Information

Below is the link to the electronic supplementary material.

Supplementary file1 (PDF 1034 KB)

## Data Availability

The data and code used during the current study are publicly available on the corresponding author’s personal github page: https://github.com/jtkerb/Nara_Paper_Repo. !Nara orthomosaic raster files are accessible at: 10.5281/zenodo.6462895.
