# Supplementary material for: Livestock impacts on an iconic Namib Desert plant are mediated by abiotic conditions
Source: Oecologia. 2022 May 7;199(1):229–42. doi: 10.1007/s00442-022-05177-w (PMC9120118; doi:10.1007/s00442-022-05177-w)
Supplement: Supplementary file 1 — Supplementary file1 (PDF 1034 KB) [file 442_2022_5177_MOESM1_ESM.pdf]

SUPPLEMENTARY MATERIALS FOR:
Livestock impacts on an iconic Namib Desert plant are mediated by abiotic conditions

Authors: Jeffrey T. Kerby[1,2,*], Flora E. Krivak-Tetley[2,3], Saima D. Shikesho[4,5], Douglas T. Bolger[2]

[1]Aarhus Institute of Advanced Studies, Aarhus University, Aarhus C, 8000, Denmark
[2] Department of Environmental Studies, Dartmouth College, Hanover, NH, 03755, USA
[3]Department of Biological Sciences, Dartmouth College, Hanover, NH, 03755, USA
[4]Gobabeb-Namib Research Institute, Namib Naukluft Park, Namibia
[5]Department of Biological Sciences, University of Cape Town, South Africa

[*]Corresponding author:
jkerby@aias.au.dk
https://orcid.org/0000-0002-2739-9096
Høegh-Guldbergs Gade 6B, 8000 Aarhus C

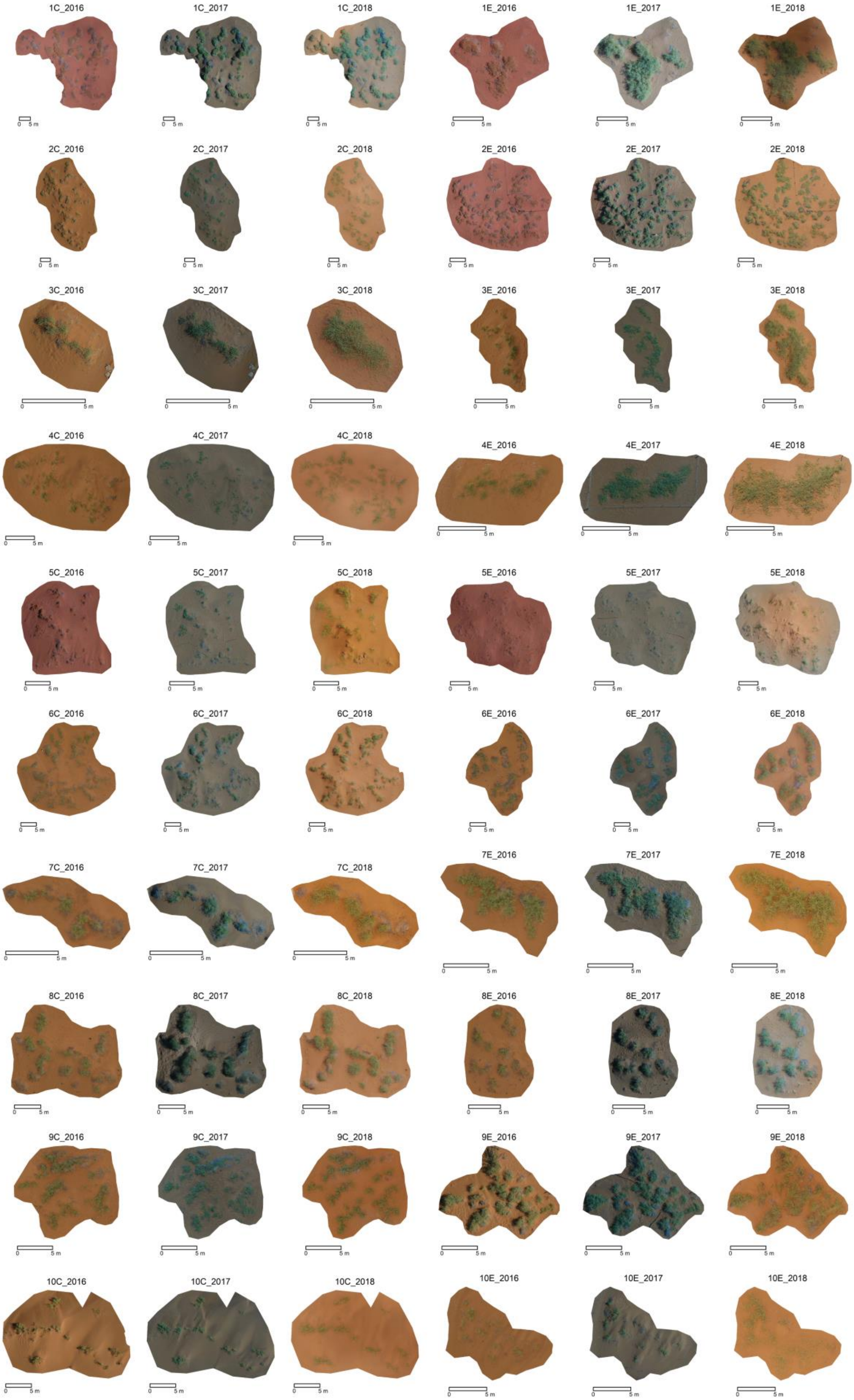
1C_2016
1C_2017
1C_2018
1E_2016
1E_2017
1E_2018
2C_2016
2C_2017
2C_2018
2E_2016
2E_2017
2E_2018
3C_2016
3C_2017
3C_2018
3E_2016
3E_2017
3E_2018
4C_2016
4C_2017
4C_2018
4E_2016
4E_2017
4E_2018
5C_2016
5C_2017
5C_2018
5E_2016
5E_2017
5E_2018
6C_2016
6C_2017
6C_2018
6E_2016
6E_2017
6E_2018
7C_2016
7C_2017
7C_2018
7E_2016
7E_2017
7E_2018
8C_2016
8C_2017
8C_2018
8E_2016
8E_2017
8E_2018
9C_2016
9C_2017
9C_2018
9E_2016
9E_2017
9E_2018
10C_2016
10C_2017
10C_2018
10E_2016
10E_2017
10E_2018

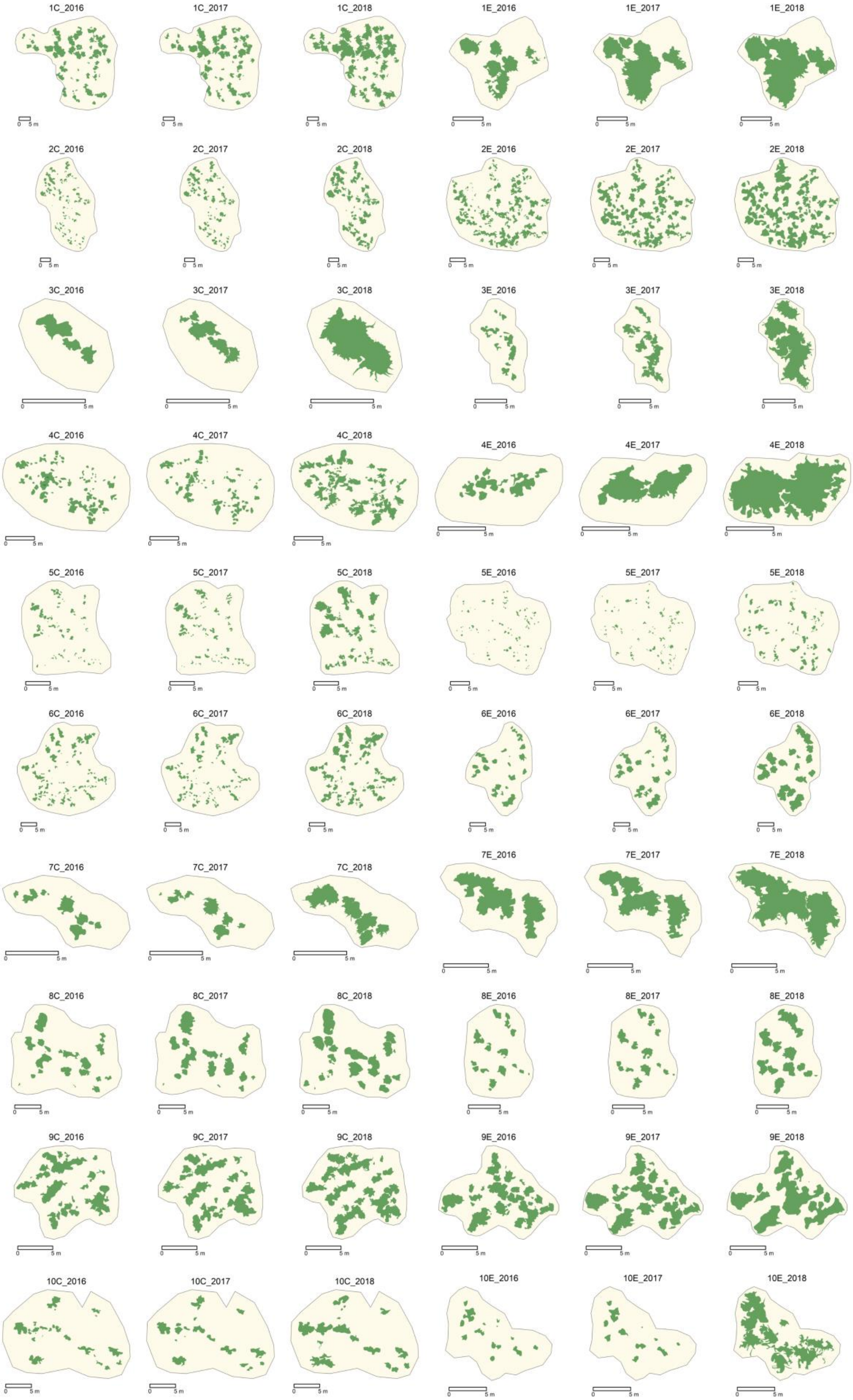
1C_2016
1C_2017
1C_2018
1E_2016
1E_2017
1E_2018
2C_2016
2C_2017
2C_2018
2E_2016
2E_2017
2E_2018
3C_2016
3C_2017
3C_2018
3E_2016
3E_2017
3E_2018
4C_2016
4C_2017
4C_2018
4E_2016
4E_2017
4E_2018
5C_2016
5C_2017
5C_2018
5E_2016
5E_2017
5E_2018
6C_2016
6C_2017
6C_2018
6E_2016
6E_2017
6E_2018
7C_2016
7C_2017
7C_2018
7E_2016
7E_2017
7E_2018
8C_2016
8C_2017
8C_2018
8E_2016
8E_2017
8E_2018
9C_2016
9C_2017
9C_2018
9E_2016
9E_2017
9E_2018
10C_2016
10C_2017
10C_2018
10E_2016
10E_2017
10E_2018
0
5 m

Supplemental Fig S1. Summary of individual drone orthomosaics and cover maps included in the data repository. The first page shows visible change in !nara condition from year to year and the second shows the measured phytomass cover (green) for each plant in each year against the background area of its hummock.

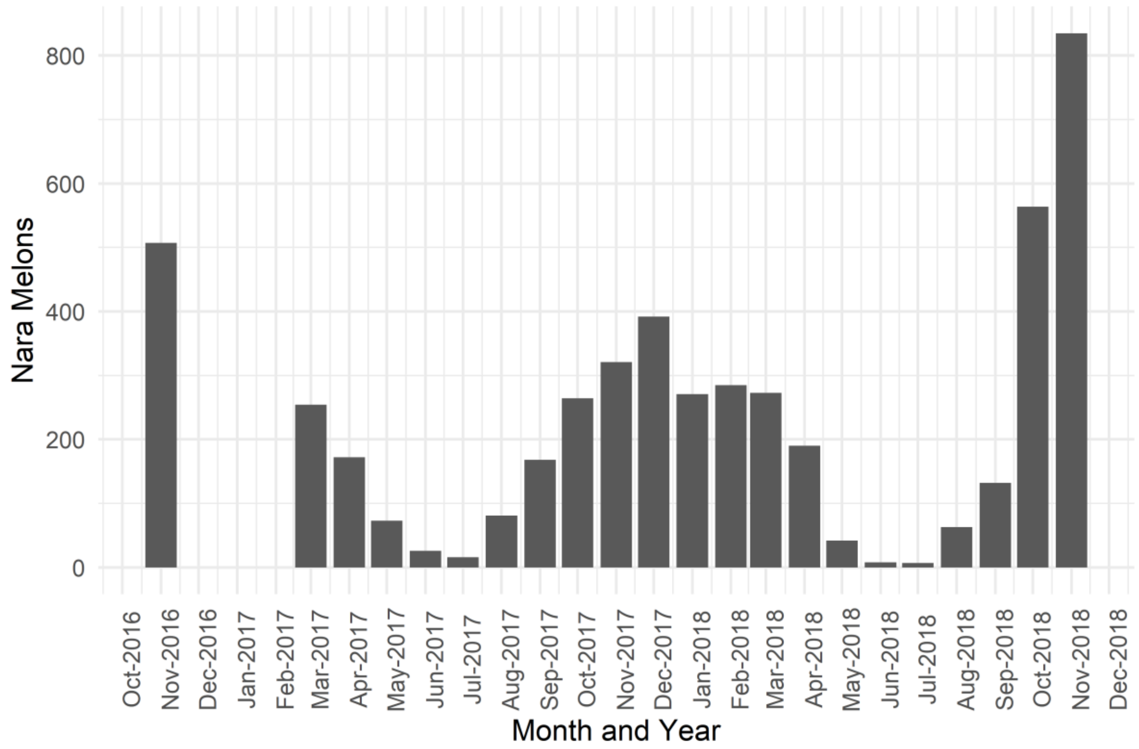

Supplemental Fig S2. Sum of all mature melons on the study plants by month showing the general peak in melon availability in the landscape around the sampling/monitoring period in late-November. As melons were counted without replacement, monthly counts are correlated and not-independent. Due to the refinement of protocols, the count from Nov 2016 also includes a limited number of mature melons of a smaller size than would be included in subsequent counts.

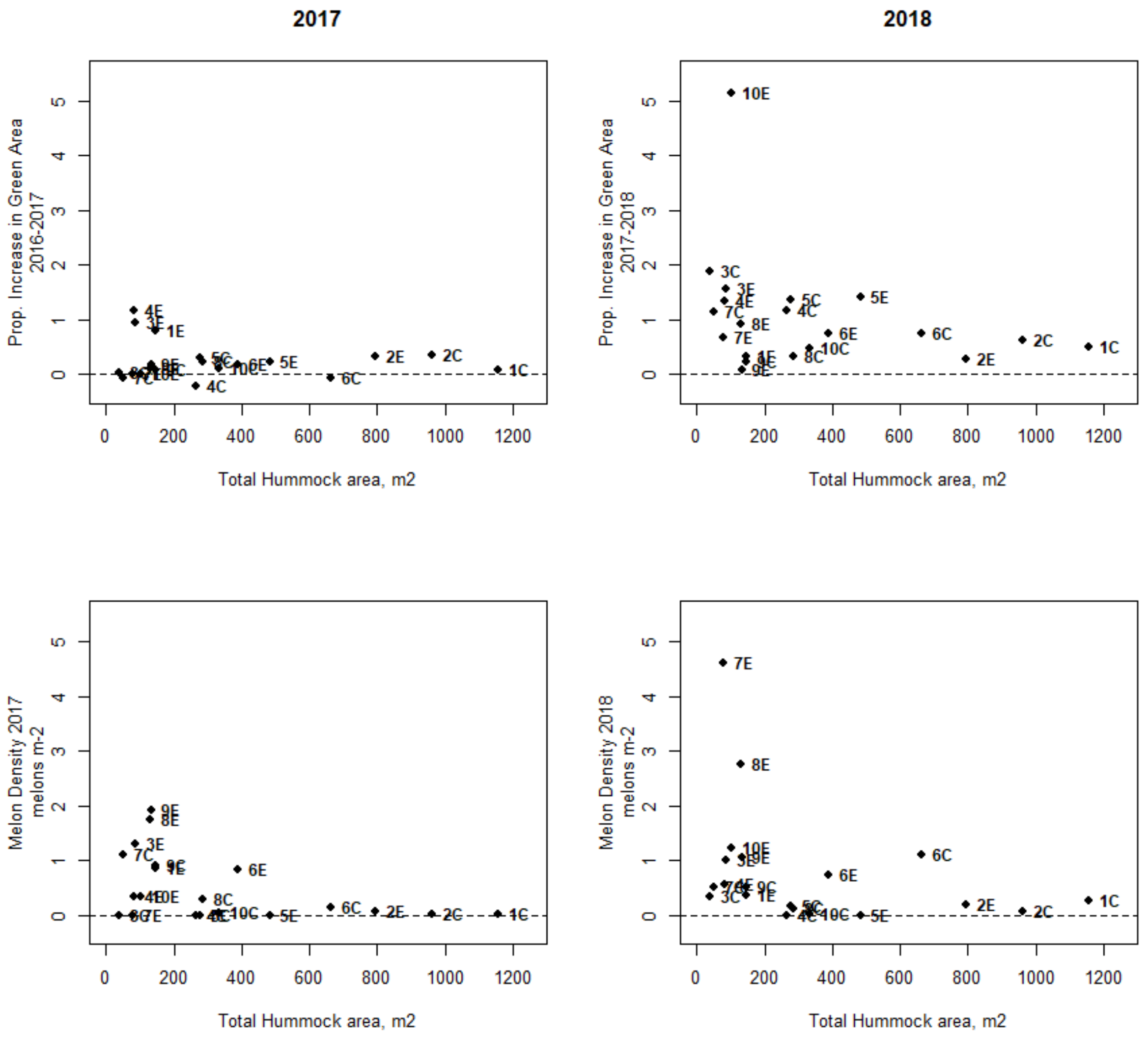

Supplemental Fig S3. Relating response variables to plant hummock size. No consistent relationship between change in green cover or melon density and hummock size is evident in either year, although there was more variability in smaller plants, especially in the wetter 2018 year. There were no clear patterns of similar responses in individual !nara in both years.
